# Towards a national trauma registry for the United Arab Emirates

**DOI:** 10.1186/1756-0500-3-187

**Published:** 2010-07-10

**Authors:** Sami Shaban, Hani O Eid, Ezedin Barka, Fikri M Abu-Zidan

**Affiliations:** 1 Trauma Group, Faculty of Medicine and Health Sciences, UAE University, Alain, UAE; 2 Department of Emergency Medicine, Tawam Hospital, Alain, UAE; 3 College of Information Technology, UAE University, Alain, UAE

## Abstract

**Background:**

Trauma is a major health problem in the United Arab Emirates (UAE) as well as worldwide. Trauma registries provide large longitudinal databases for analysis and policy improvement. We aim in this paper to report on the development and evolution of a national trauma registry using a staged approach by developing a single-center registry, a two-center registry, and then a multi-center registry. The three registries were established by developing suitable data collection forms, databases, and interfaces to these databases. The first two registries collected data for a finite period of time and the third is underway. The steps taken to establish these registries depend on whether the registry is intended as a single-center or multi-center registry.

**Findings:**

Several issues arose and were resolved during the development of these registries such as the relational design of the database, whether to use a standalone database management system or a web-based system, and the usability and security of the system. The inclusion of preventive medicine data elements is important in a trauma registry and the focus on road traffic collision data elements is essential in a country such as the UAE. The first two registries provided valuable data which has been analyzed and published.

**Conclusions:**

The main factors leading to the successful establishment of a multi-center trauma registry are the development of a concise data entry form, development of a user-friendly secure web-based database system, the availability of a computer and Internet connection in each data collection center, funded data entry personnel well trained in extracting medical data from the medical record and entering it into the computer, and experienced personnel in trauma injuries and data analysis to continuously maintain and analyze the registry.

## Background

### Trauma worldwide and in the UAE

Trauma is the cause of 10% of all deaths worldwide [1] and it is projected that road traffic deaths will increase by 83% between 2000 and 2020 in developing countries [2]. Trauma is a major health problem in the United Arab Emirates (UAE). About 18% of the annual mortality in the UAE is due to trauma and most of these are caused by road traffic collisions [3]. Trauma affects mainly the young productive population, which has a profound health and economical impact. Prevention of trauma is the most cost-effective method for reducing the death toll [4].

### Trauma registries

One factor for potentially preventing trauma is to monitor it through trauma registry surveillance systems [5]. Trauma registries are databases that document trauma cases according to specific inclusion criteria [6]. They are designed to improve injury surveillance and enhance trauma care, outcomes, and prevention [4]. It has been shown that trauma registries in developing countries are plausible and valuable tools for injury surveillance [4,5].

### Benefits of trauma registries

Trauma registries are valuable tools for identifying trauma areas that require quality improvement policy implementation, and they can be of benefit to the progress of the health care system [7]. Trauma registries may also give insight into the nature and extent of trauma [4,6], and play an important role in monitoring the management of injured patients [8]. In addition, trauma systems improve survival rates of injured patients [9] and trauma registries are an integral part of these systems. Countries with limited resources have been able to establish useful Trauma registries [4,8,10,11]. A trauma registry provides a large and longitudinal database for analysis [5]. This yields more reliable information regarding risk factors related to different types of injuries and possible preventive measures. For example, a German group used their trauma registry to successfully study prehospital triage and survival of major trauma patients [12].

### Technical development of trauma registries

Technically, trauma registries are simply databases. Since relational databases are the most popular design for databases today due to their simplicity and reliability [13], it follows that trauma registry databases are developed by using relational database design. As with any database system, the user interface design is extremely important, and even more so with trauma registry interfaces due to medical terminology and trauma score complexities. Data entry users of the registry must be well trained in dealing with the registry's interface as well as in dealing with the patients' medical records. This study is not an enhancement in relational database theory and design, but rather an application in the correct use of relational databases for developing trauma registries.

There are several articles which discuss various aspects of registry development. In Australia, they conclude that developing a robust, easy to use and integrated database management system is difficult and requires skilled personnel. In addition, linking the databases for a statewide registry is particularly difficult because of incompatible systems across hospitals [14]. In Haiti, where a hospital-based trauma registry was established using a task force of medical personnel and IT technicians, it was concluded that it is possible to improve injury surveillance in developing and resource-poor settings [4]. The same was stated in Nigeria when developing their own trauma registry while emphasizing the importance of designing a data set for the registry which is valid, reliable, and possible to collect efficiently [10].

The Chinese Maxillofacial Trauma Registry was developed as a tool to collect epidemiological characteristics, treatment, and outcome [15]. They developed a web-based database-driven model, similar to our multi-center model, which utilizes the web, client computers, and a central database server to store the data. As with previous registries, they also had to consider a specific set of data elements to collect. In addition, they had to consider backup and security issues because of their distributed approach. They concluded that their system has satisfactory stability, security, compatibility, and specialty; and that it sufficiently collects data from standard cases of maxillofacial trauma.

A US army trauma surgical group developed a trauma registry in a forward deployed military unit [16]. They utilized Personal Digital Assistants (PDA handheld devices) to collect data and send it to a Microsoft (MS) Access database on a portable laptop computer. They conclude that this method can be an efficient and effective method in expanding trauma registries in forward deployed surgical units. Finally, Protetch and Chappel discuss the process of developing, implementing, and refining a registry data validation system in order to achieve optimal trauma registry operations [17].

### Aim of study

We aim in this paper to report on the development and evolution of a national trauma registry. We took a staged approach by developing a single-center registry first then a two-center registry and, finally, a multi-center trauma registry, learning valuable lessons as we progressed. In addition, we were able to determine the factors and obstacles in establishing a multi-center registry so as to serve as a model of nationwide Health Informatics research.

## Methods

### Al-Ain Hospital trauma registry

The steps involved in the establishment of the trauma registry at Al-Ain Hospital were the design of a suitable data entry form, definition of inclusion/exclusion criteria, supplying computer hardware and software (MS Access database), and securing of salary for the trauma research assistant from a grant (see Acknowledgement).

With regard to the trauma registry database, the decision was made to use MS Access 2000 as the standalone database system due to its ease of use, quick development style, support for relational database design, support for forms and reports, and availability.

Developing the database based on the paper form was rapid. Feedback from the Trauma Group (UAE University) and the data entry research assistant was collected and modifications were made. The database was piloted by the trauma research assistant on several relevant patient cases. Some shortening of the form took place at this stage to minimize data entry length.

Most of the design and modification issues centered around field data types and values for dropdown lists. The database was designed with a main menu and buttons for each of the forms. A non-tabbed form was used for this database because it was found by the users to be easier to use at the time.

### Two-center road traffic collision injury registry

This registry extended data collection to involve the two main hospitals in Al-Ain City, Al-Ain and Tawam Hospitals, but limited the data collection form further to include only road traffic collision injuries. A precise and limited data collection form was designed. A web-based database was developed and data were entered into it. Data were collected prospectively for 18 months, from April 2006 to October 2007.

Although the computer system was web-based, it was installed and used on a laptop. The laptop ran Apache web server with MySQL database. The website was written in PHP web programming language. The data entry research assistant was forced to use the system as a standalone system because of lack of Internet connectivity at the time. The database design was essentially a subset of the original trauma database; however, all patient data was stored in a single table.

### Multi-center Trauma Registry starting in Tawam Hospital

Establishment of the multi-center trauma registry followed these steps:

I. Modification of the registry form: The old form was shortened from 7 to 4 pages to include only essential variables.

II. Inclusion and exclusion criteria were defined.

III. Database development: MS Access was used initially to collect, store, and maintain the data on a single computer. Then, the Access database was converted into an MS SQL Server database using ASP as the web programming language to facilitate multi-center data collection.

IV. Data entry: Selecting, training, and funding of one key personnel for data entry in one collection center was established. We are awaiting funding for data entry personnel in each center.

V. Data analysis and reporting: A team of trauma experts and a data analyst are performing continuous data analysis and reporting of the trauma registry.

The first phase of database development involved the design of an MS Access 2007 database containing items from the developed paper form. In contrast to the previous non-tabbed Access database, a tabbed approach was used for developing a single tabbed form with a single patient data table which users now preferred. Development was quick as we had the experience of previous database registries.

## Results

### Al-Ain Hospital trauma registry

2,573 patients were registered over a three-year period starting from 2003. The data entry research assistant was satisfied with the design and performance of the Access database. The main critique was the inconvenience of having each section of the patient's information open in a separate window. Queries were easily developed for analysis and reports. In total, 13 forms were designed, as well as two reports and over 25 queries used to generate the reports and progress indicators. Twelve tables were created which are related one-to-one with two lookup tables related one-to-many.

### Two-center road traffic collision injury registry

1008 patients were entered into the registry prospectively for 18 months, from April 2006 to October 2007. The inability to use the database as an online web-based application due to lack of Internet connectivity at the time proved to be a minor issue. That was because only two centers, which are located in the same city, were included. It was not difficult for the data entry research assistant to carry a laptop and move between hospitals.

### Multi-center trauma registry starting in Tawam Hospital

Data collection has started for the first phase which includes Tawam Hospital, with plan to include more hospitals soon. The refined Access database is being used for data entry while the web-based application is being developed. Piloting of the tabbed Access database was highly successful.

## Discussion

From these experiences in establishing trauma registries, we have learned that a knowledgeable data entry person is vital to the success of the registry due to the fact that accurate and correct data entry is one of the most crucial aspects of registries. An Australian group reached the same conclusion when developing their own statewide trauma registry [14]. They state that an interdisciplinary group of well trained and skilled researchers and clinicians must collaborate to collect and analyze the data.

In all three registries, medically trained research assistants were in charge of prospectively collecting the required data on a daily basis from the patients and their medical records, then entering them into the registry's database. The research assistants gradually became experienced in data extraction and entry. This daily prospective collection increased our confidence on the completeness and quality of the data in the registry. Almost all patients who met the inclusion criteria were entered into the registry and specific field missing values were kept as minimum as possible. On several occasions data in the registry were checked against the medical records to identify defects needing action.

**Figure 1:**
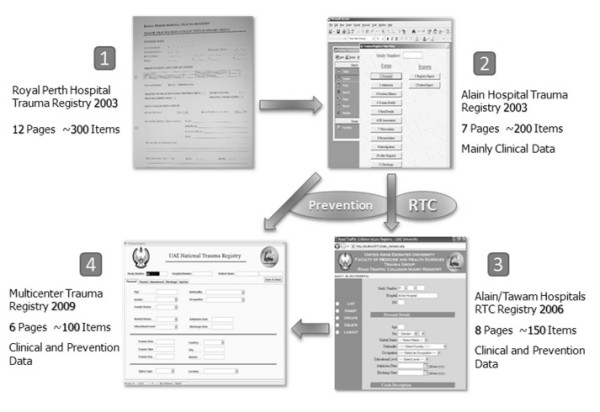
**Evolution of the trauma registry in the UAE**.

We benefited from the staged approach in not having to acquire all funds at once. We were also slowly able to refine our data form and reduce the total number of data elements collected while adding data elements important for preventive medicine. Figure 1 shows the evolution of the trauma registries in the UAE. Other countries such as the USA, Germany, and Australia are in the same situation where they have local trauma registries and are struggling with establishing a nation-wide trauma registry [8,12,18].

Taking a public health approach in the design of trauma registries adds a level of completeness to the research. Including preventive medicine researchers from the beginning as well as the addition of select data elements that have public health implications is important. We learned this after the first trauma registry and therefore included several public health measures in the second and third registries, such as items related to preventative medicine and road traffic injuries (Figure 1). The impact of this research on public health policy is evident by the partial influence the trauma registry has had on raising trauma health issues in the country related to occupational injuries and road traffic collisions. In particular, the need for prevention of occupational injuries and the legislation regarding the need for child and rear seatbelts in the UAE are highlighted in several newspaper articles appearing in 2009 in one of the country's leading newspapers, The National [19-24].

Identifying factors and obstacles involved in establishing a trauma registry is an important step towards the success of the registry. Many obstacles faced when conducting research in developing countries have been identified in the literature [10,25]. These include lack of research education, lack of appreciation of the value of research, and shortage of funding, among others. From our experiences, we have identified the following common obstacles:

1. Lack of appreciation of the value of database registries.

2. Hardships in securing funds for this kind of research.

3. Hiring and training good data entry research assistants.

4. Lack of Health Informatics experts.

5. Lack of harmony between researchers in multiple disciplines.

6. Lack of motivation for doctors to participate in such research.

These obstacles are exaggerated in the case of a multi-center national trauma registry due to the existence of additional logistics such as remote data entry and protection of patient privacy over the Internet. Establishing a secure online data collection method is based on the web-based database model. This data collection, storage, and maintenance model is used to perform secure data collection over the Internet. It uses a centralized database on the server side, which allows for real-time data collection and storage. Data security can be taken into account in the following ways:

1. Username and Password Protected Web Site: Only authorized data entry personnel are allowed to enter the data entry web site.

2. Encrypted data transfer: Data are transferred from the web browser to the server in an encrypted and secure fashion.

3. Username and password protected data browsing and editing: Only authorized research personnel are allowed to view and edit the data in the registry, each according to his or her privileges.

4. Logging: All access to and activity on the registry is logged. The log is accessible to authorized data managers only and contains client username, client IP address, date and time stamp, records accessed, and activity.

5. Username and password protected server: The computer server on which the data resides is also protected by username and password, and only authorized technicians and data managers are given access.

The adaptability of these registries in other countries is of great importance. In order to export our experiences to other countries these registries must be easy to install and use. The first trauma registry in Al-Ain Hospital and the third trauma registry started in Tawam Hospital were both developed using MS Access which is easy to transport and modify to suit a specific country's needs. The second registry (Road Traffic Collision Injury Registry) is more difficult to transport and install because it uses a web-based system.

Once hospitals in the UAE establish a working Electronic Medical Record (EMR) system, many data items in the registry can be directly imported from the EMR. This will cut down on the tedious data entry process for these registries. The registry database would have to be adapted to accommodate the ICD coding used by the EMR. The EMR also benefits from trauma registries that use standard injury severity measurements such as the Trauma Score, the Glasgow Coma Scale (GCS), and the Injury Severity Score (ISS). These measures are implemented in many trauma registries [4-7,10] and have been implemented in our systems as well.

In summary, we hope to have shown that trauma registries are essential tools for quality management over an extended period of time and for integrating primary, secondary and tertiary prevention strategies into injury control. In addition, the implementation of trauma registries is an iterative and difficult process that requires buy-in from a number of constituencies whose appreciation increases with their investment and perception of added value to their individual missions.

Finally, the usefulness of registries is apparent by the extent in which data is requested from them and research articles are published based on their findings. In the case of the first two registries, several publications have emerged [26-33].

## Conclusion

Establishing a multi-center national trauma registry in a developing country is a formidable task. It is advisable that the following be accomplished in order to get the most benefit from the registry:

1. Development of a suitable and concise registry data entry form.

2. Development of a database and website translating the registry data entry form into a secure online electronic form.

3. Availability of a computer and Internet connection in each data collection center.

4. Funded data entry personnel in each data collection center who are well trained in extracting wanted medical data from the medical record and entering them into the web form.

5. Experienced personnel in trauma injuries and data analysis in order to continuously maintain and analyze the registry.

We have learned during the three phased approach that, first, the inclusion of preventive medicine researchers and data elements is important in any trauma registry in order to understand how to better prevent injuries, and second, the focus on Road Traffic Collision data elements is essential in a country such as the UAE due to high percentage of RTCs in the UAE which involve UAE nationals.

Developing the registry database as a standalone system (i.e. MS Access) or as a web-based system depends on the ultimate purpose of this database. If there is a need to transport the database to other countries so that they may establish their own trauma registry then a simple standalone database is preferred. On the other hand, if the purpose is to establish a nationwide multi-center trauma registry then a web-based model is preferred.

## Competing interests

The authors declare that they have no competing interests.

## Authors' contributions

SS helped in the idea and design of the first and third trauma registry forms, designed and developed the electronic trauma registries, analyzed the data, and wrote and edited the manuscript. HE helped in the idea, collected and entered the data. EB helped in the idea and design of the second trauma registry form, designed and developed the electronic trauma registry. FMA had the idea, raised funds for the study, designed the trauma registry forms, trained the research fellows in data collection, assured the quality of data collected, did the primary analysis, helped draft the first version of the paper and repeatedly edited it. All authors read and approved the final manuscript.
